# Epigenetic Clock Analysis of Sex Chromosome Aneuploidies

**DOI:** 10.1111/acel.70243

**Published:** 2025-09-30

**Authors:** Joshua Zhang, Jordan Teoli, Benjamin Rey, Cristina Vieira, Jean‐François Lemaitre, Ingrid Plotton, Gabriel A. B. Marais, Steve Horvath

**Affiliations:** ^1^ Department of Human Genetics, David Geffen School of Medicine at UCLA University of California Los Angeles Los Angeles California USA; ^2^ Service de Biochimie et Biologie Moléculaire, Unité Médicale de Biologie Endocrinienne, Centre de Biologie et Pathologie Est Hospices Civils de Lyon Bron France; ^3^ Université Claude Bernard Lyon 1 Lyon France; ^4^ Laboratoire de Biométrie et Biologie Evolutive, UMR 5558, CNRS Université Claude Bernard Lyon 1 Villeurbanne France; ^5^ Institut Cellule Souche et Cerveau (SBRI), Unité INSERM 1208 Centre de Recherche INSERM Bron France; ^6^ Service de médecine de la reproduction Hôpital Femme‐Mère‐Enfant, Hospices Civils de Lyon Bron France; ^7^ Department of Biostatistics, Fielding School of Public Health University of California Los Angeles Los Angeles California USA; ^8^ Altos Labs Cambridge Cambridgeshire United Kingdom

**Keywords:** aneuploidy, DNA methylation, epigenetic clock, gonosome, Klinefelter syndrome, X chromosome

## Abstract

Sex differences in lifespan are pervasive in nature and in humans, yet the contribution of sex chromosomes to DNA methylation–linked aging remains incompletely defined. We leveraged human sex chromosome aneuploidies to test whether X and Y chromosome dosage influences epigenetic aging, quantified with DNA methylation (DNAm) clocks. In whole blood from individuals with karyotypes 46,XX (female) and 46,XY, 47,XXY, 47,XYY (male), we measured epigenetic age and age acceleration using a first‐generation clock (Skin & Blood) and two next‐generation clocks (GrimAge and DunedinPACE), and examined the components underlying GrimAge. Next‐generation clocks indicated lower epigenetic age acceleration and a slower pace of aging in 47,XXY versus 46,XY. In GrimAge, a weighted sum of DNAm surrogates, higher DNAmLeptin (negatively weighted) and lower DNAmPACKYRS (positively weighted smoking proxy) in 47,XXY jointly reduced GrimAge in comparison to 46,XY. DunedinPACE also indicated a slower pace of aging in 47,XXY. In contrast, the first‐generation Skin & Blood clock indicated higher age acceleration in 47,XXY and 47,XYY than in 46,XY. Enrichment analyses of autosomal EWAS loci highlighted immune pathways and karyotype‐specific signatures, for example, metabolic/cancer‐related processes in 47,XXY and renal/amino acid transport processes in 47,XYY. Overall, X chromosome gain in 47,XXY was associated with lower GrimAge and slower DunedinPACE, whereas the Skin & Blood clock diverged, suggesting that DNAm clocks capture partially distinct biological domains of aging differentially perturbed by sex‐chromosome dosage. These findings motivate larger, age‐spanning studies with direct phenotyping to define mechanisms.

## Graphical abstract

Next‐generation epigenetic clocks indicate lower age acceleration and slower pace of aging in 47,XXY than 46,XX, 46,XY, and 47,XYY. A first‐generation clock (Skin & Blood) indicates higher age acceleration in 47,XXY and 47,XYY than 46,XY, while higher naïve CD8^+^ T in 47,XXY than both 46,XY and 46,XX suggests reduced immunosenescence.

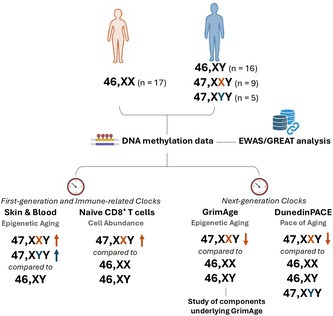

## 1 Introduction

Differences in life expectancy between the sexes are particularly pervasive in the living world (Marais et al. 2018; Bronikowski et al. 2022), and humans are no exception. Though it varies between countries, females consistently live longer than males (Feraldi and Zarulli 2022), with a mean difference in life expectancy at birth of about 5 years (World Health Organization 2025). As a consequence, over 90% of supercentenarians are women (Robine and Vaupel 2001).

Different theories have been proposed to explain this consistent feature. Among them, earlier theories have invoked a role of sex chromosomes (Lemaître et al. 2024). Prior hypotheses predicted that men carrying only one copy of the X chromosome would consequently be more sensitive to deleterious recessive mutations occurring on their single X copy, also known as the unguarded X hypothesis (MacArthur and Baillie 1932; Connallon et al. 2022; Ostan et al. 2016; Bonduriansky et al. 2008; Lemaître et al. 2024 ). Recently, mechanisms behind the influence of sex chromosome on the sex differences in lifespan have been totally revisited with a new focus on the Y chromosome and its potential toxicity for males, also known as the toxic Y chromosome hypothesis (Brown et al. 2020; Teoli et al. 2025), which occurs due to various detrimental genetic and physiological pathways (reviewed in Marais et al. 2018).

Additional theories, not related to the sex chromosomes, have put forward the influence of other mechanisms. This is notably the case of the mother's curse theory, arguing for an accumulation of mutations with men‐specific deleterious effects across the mitochondrial genome (Camus et al. 2012; Maklakov and Lummaa 2013; Milot et al. 2017). In addition, the sex differences in life history strategy theory (Trivers 1972) emphasize that sexual selection pressures have favored the evolution of a “live fast—die young” strategy in males, underpinned by the endocrine system (Brooks and Garratt 2017).

In humans, there is evidence that sexual hormones, such as exposure to testosterone (Schooling and Zhao 2021), mother's curse effects (Milot et al. 2017), as well as additional cultural and environmental differences between men and women (Luy 2003) such as differences in work (Bahu et al. 2013), in diet (Razaz et al. 2022; Bärebring et al. 2020), or in exposure to risky behaviors (Kaplan and Erickson 2000; Wilsnack et al. 2000), all contribute to establishing the gap in lifespan between sexes (Austad and Fischer 2016; Marais et al. 2018).

The contribution of sex chromosomes on sex‐specific lifespan in humans is yet to be thoroughly explored. Studying sex chromosome aneuploidies in humans is a way to evaluate how sex chromosomes contribute to sex differences in longevity. Demographic data from patients with sex chromosome aneuploidies point to a possible toxic Y effect, as they suggest a reduction in lifespan of approximately 10 years in 47,XYY individuals known as Jacob syndrome patients (Stochholm et al. 2010), while 47,XXY individuals known as Klinefelter syndrome patients hold a reduction in lifespan of approximately 2 years (Bojesen et al. 2004, 2011).

DNA methylation at CpG sites changes over time, allowing for the construction of “epigenetic clocks” that show a strong correlation between epigenetic age and chronological age (Horvath 2013; Bell et al. 2019). The first‐generation clocks, which are designed to estimate chronological age, demonstrate strong positive correlations between epigenetic age and chronological age. This includes the pan‐tissue clock (Horvath 2013), the Skin & Blood clock (Horvath et al. 2018), and the blood‐based clock introduced by Hannum in 2013 (Hannum et al. 2013). In a similar vein, second‐generation clocks, which aim to estimate human mortality risk, such as PhenoAge (Levine et al. 2018) and GrimAge (Lu, Quach, et al. 2019), also exhibit strong positive correlations with age. Chronological age exhibits a negative correlation with the DNA methylation‐based estimate of telomere length (Lu, Seeboth, et al. 2019). Telomere shortening is a well‐known hallmark of cellular senescence and aging (Vaiserman and Krasnienkov 2021).

The DunedinPACE estimator is only weakly correlated with chronological age (typically ranging from *r* = 0.2 to 0.5). Clocks like GrimAge, which integrate multiple DNAm surrogates for plasma proteins and smoking pack‐years, show high predictive power for incident disease and all‐cause mortality (Lu, Quach, et al. 2019; Lu et al. 2022).

Chronological age can confound associations between epigenetic biomarkers and aneuploidy status. To remove this age effect, we use epigenetic age acceleration (EAA), defined as the residual from a regression of the epigenetic age estimate (outcome) on chronological age. EAA therefore quantifies how much an individual's epigenetic age deviates from what is expected given their chronological age. Higher EAA has been linked to increased risk of age‐related diseases (Bell et al. 2019; Saul and Kosinsky 2021) and shorter time to death (Chen et al. 2016; Horvath et al. 2015; Marioni et al. 2015; Zhang et al. 2017).

In this study, we examined widely used epigenetic clocks in 46,XX women and 46,XY, 47,XXY, and 47,XYY men along with additional individuals with differences of sex development (DSD) (46,XY individuals with a complete androgen insensitivity syndrome [CAIS] who are phenotypically females, 46,XX SRY positive individuals who are phenotypically males) to test if epigenetic age depends on the sex chromosome composition of the karyotype. Our predictions were that 47,XXY and 47,XYY men should exhibit greater EAA rate than 46,XY men, with the greatest acceleration for 47,XYY owing to the demographic data mentioned above (Stochholm et al. 2010; Bojesen et al. 2004, 2011).

Strikingly, 47,XXY individuals show significantly lower GrimAge age acceleration and lower DunedinPACE values (Belsky et al. 2022) than other individuals (notably 46,XY men) consistent with a slower biological aging process in 47,XXY. To identify drivers of this difference, we decompose GrimAge into its component measures and assess their contributions to the reduction in GrimAge acceleration. Finally, we conduct an epigenome‐wide association study (EWAS) and annotate the resulting loci using GREAT (Genomic Regions Enrichment of Annotations Tool).

## 2 Results

### 2.1 Epigenetic Age Estimates Correlate Strongly With Chronological Age

**FIGURE 1:**
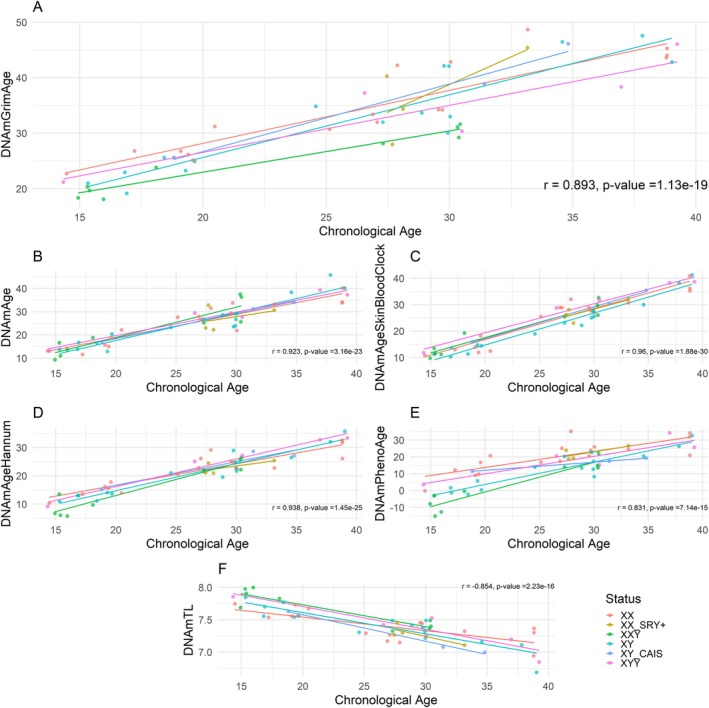
Epigenetic age estimate versus chronological age. (A) GrimAge clock, (B) Pan‐tissue clock, (C) Skin & Blood clock, (D) Hannum clock, (E) PhenoAge clock, (F) Telomere clock. Each line represents one group: XX (*n* = 17), XX SRY+ (*n* = 4), XXY (*n* = 9), XY (*n* = 16), XY CAIS (*n* = 3), and XYY (*n* = 5). The colored lines are from a linear regression of epigenetic age on chronological age performed in each group. The correlation and *p* value for each graph was calculated for all samples regardless of group.

Our analysis reveals that the first‐generation and second‐generation epigenetic clocks exhibit the anticipated strong positive correlations with chronological age at the time of blood draw (Figure 1). Furthermore, chronological age exhibits a strong negative correlation with the DNA methylation‐based estimate of telomere length (Figure 1).

### 2.2 GrimAge: Epigenetic Age Is Lower in 47,XXY Than 46,XY and 46,XX

Because GrimAge includes sex as a covariate, we held sex constant by setting it to “female” (constant value 1) for all samples, isolating karyotype effects on GrimAge and its components (such as DNAmLeptin). This effectively removed GrimAge's dependency on sex, allowing us to isolate the variables of primary interest.

**FIGURE 2:**
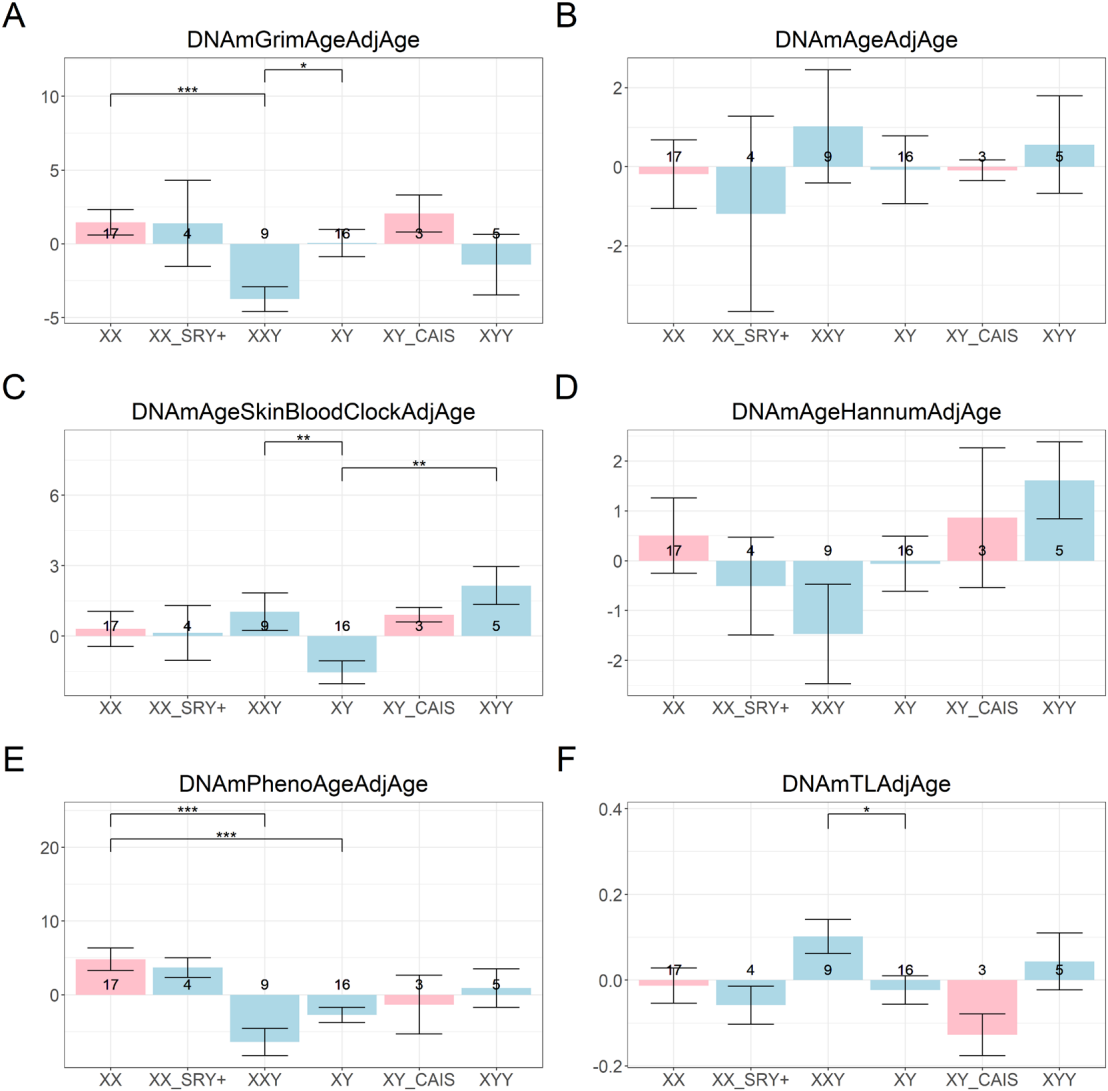
Epigenetic age acceleration versus group. The *y*‐axis shows age‐adjusted (“AdjAge”) estimates (“age acceleration”) of the (A) GrimAge clock, (B) Pan‐tissue clock, (C) Skin and blood clock, (D) Hannum clock, (E) PhenoAge clock, and (F) Telomere clock. The groupings/bars correspond to XX females, XX SRY+ phenotypic males, XXY males, XY males, XY CAIS phenotypic females, and XYY males. The pink bars represent phenotypic females and the light blue bars represent phenotypic males. Each bar plot depicts the mean values (*y*‐axis) along with one standard error. The significance brackets are displayed for select biologically relevant comparisons (see Section 5). The asterisks above the significance brackets correspond to significance levels from a Wilcoxon rank‐sum test of *p < 0.05, **p < 0.01, and ***p < 0.001. Each bar plot depicts the mean values (*y*‐axis) along with one standard error. Sample sizes (counts) are reported in each bar. We used the R package ggsignif (Ahlmann‐Eltze and Patil 2021).

The GrimAge clock suggests that 47,XXY males are epigenetically younger than both 46,XY males and 46,XX females (*p* < 0.05 and *p* < 0.001, respectively; Figure 2). Similarly, the age‐adjusted methylation‐based estimate of telomere length (the “Telomere methylation clock”) indicates that 47,XXY males have longer telomere length estimates compared to 46,XY males (*p* < 0.05), further supporting the notion of a younger methylome in these individuals (Figure 2).

In contrast, the Skin & Blood Clock indicates that 47,XXY males and 47,XYY males are epigenetically older than 46,XY males (both *p* < 0.01). This highlights a key discrepancy: second‐generation clocks such as GrimAge yield conclusions about 47,XXY males that differ from those drawn using first‐generation clocks, that is, the Skin & Blood Clock. The results for DunedinPACE are presented below.

### 2.3 DNAm‐Based Components Underlying GrimAge

The GrimAge clock is defined as a linear combination of eight DNAm‐based components that estimate plasma proteins and smoking pack‐years. Components with negative weights lower the GrimAge estimate when increased, whereas positively weighted components raise it. With the exception of DNAmLeptin, all components enter the GrimAge model with a positive coefficient. We examined which of these eight DNAm‐based components differ across karyotypes after adjusting for chronological age and holding sex constant as a covariate. With the exception of DNAmB2M, seven components showed nominally significant differences in various pairwise comparisons. We summarize the main findings from this component analysis in the following.

**FIGURE 3:**
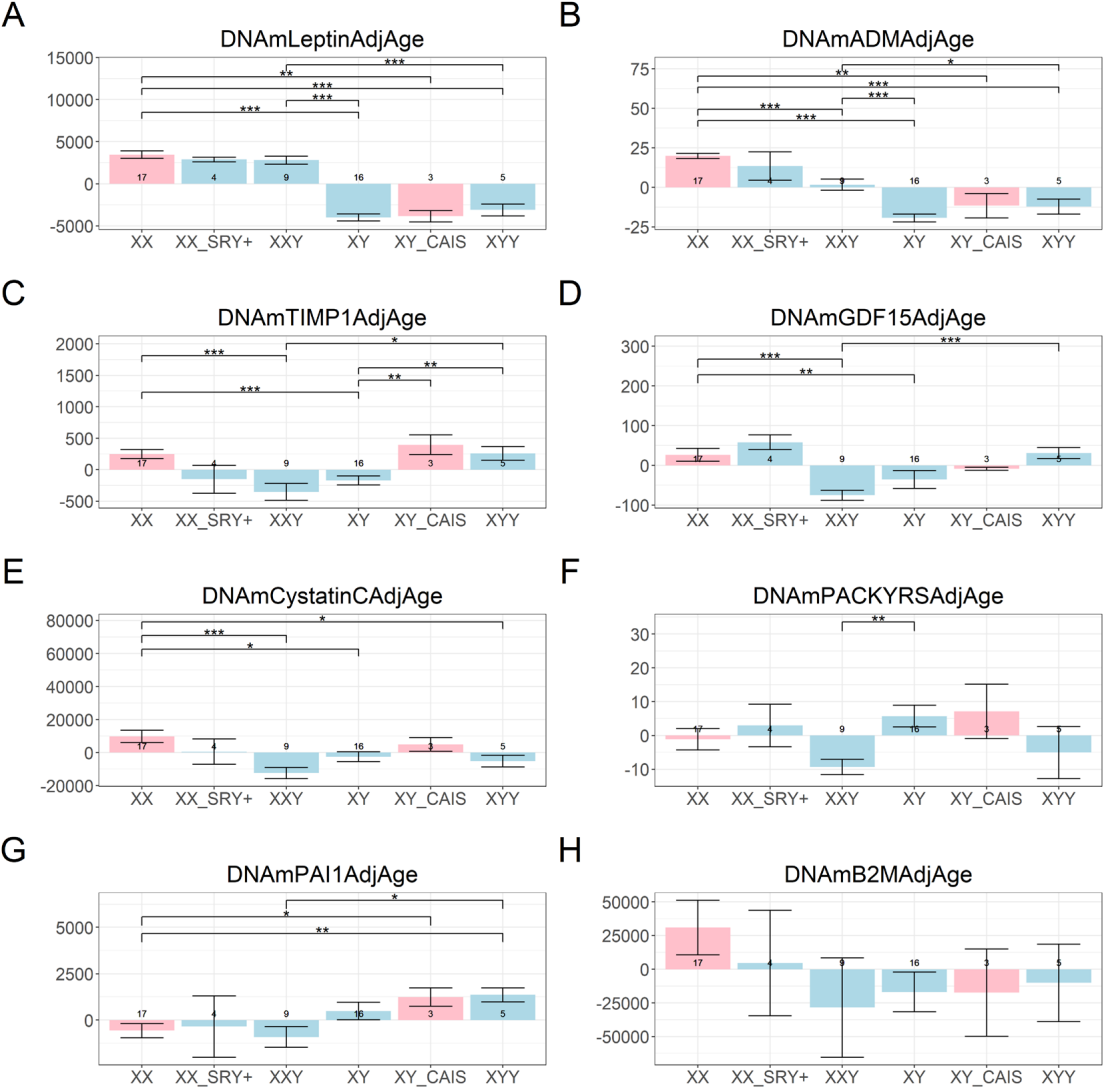
Components of the GrimAge Clock. Each bar plot corresponds to age‐adjusted (“AdjAge”) DNAm‐based surrogates of (A) leptin, (B) adrenomedullin levels, (C) tissue inhibitor of metalloproteinase 1, (D) growth differentiation factor 15, (E) cystatin C, (F) smoking pack‐years, (G) plasminogen activator inhibitor 1, and (H) beta‐2 microglobulin. The groupings/bars correspond to XX females, XX SRY+ phenotypic males, XXY males, XY males, XY CAIS phenotypic females, and XYY males. The pink bars represent phenotypic females and the light blue bars represent phenotypic males. The significance brackets are displayed for select biologically relevant comparisons (see Section 5). The asterisks above the significance brackets correspond to significance levels from a Wilcoxon rank‐sum test of *p < 0.05, **p < 0.01, and ***p < 0.001. These DNAm‐derived measures reflect ordinal values and do not have any units. Each bar plot depicts the mean values (*y*‐axis) along with one standard error. Sample sizes (counts) are reported in each bar.

First, both DNAmLeptin and DNAmADM levels were significantly higher in individuals with two X chromosomes (46,XX; 46,XX SRY+; 47,XXY) compared to those with a single X chromosome (46,XY; 46,XY CAIS; 47,XYY) (*p* < 0.001, Figure 3A,B). High DNAmADM (adrenomedullin) levels are predictive of mortality risk, whereas prior studies have shown that DNAmLeptin does not positively predict mortality (Lu, Quach, et al. 2019). Second, the methylation‐based smoking proxy, DNAmPACKYRS, was significantly lower in 47,XXY males compared to 46,XY males (*p* < 0.01, Figure 3F). This may partially reflect the lower prevalence of smoking among 47,XXY individuals (22.2%, *n* = 9) relative to 46,XY individuals (37.5%, *n* = 16; Tables 1 and 2).

**TABLE 1:** Sample counts by group.

Group	Sex phenotype	Number of individuals	Number of technical replicates (if any)	Mean age ± SD years	Number of known current smokers (n individuals)
46,XX	Female	17	3/17 individuals have duplicates	26.8 ± 7.7	1
46,XY	Male	16	6/16 individuals have duplicates	26.2 ± 7.7	6
47,XXY	Male	9	9/9 individuals have duplicates	22.0 ± 7.3	2
47,XYY	Male	5	5/5 individuals have triplicates	29.5 ± 9.9	2
46,XY CAIS	Female	3	3/3 individuals have triplicates	28.4 ± 6.9	0
46,XX SRY+	Male	4	2/4 individuals have triplicates 1/4 individual has duplicates	29.1 ± 2.7	3

Abbreviations: CAIS, complete androgen insensitivity syndrome; SD, standard deviation.

**TABLE 2:** Characteristics of 47,XXY individuals.

Individual no.	Chronological age	Karyotype	Smoking status	Body mass index (kg/m^2^)	Bone mineral density	Testosterone (nmol/L) (reference range: 10.4–26.0)	Bioavailable testosterone (nmol/L) (reference range: 2.25–10.70)	Ongoing treatment	History
1	27.31	47,XXY (XXY in 95% of mitoses, XXXY in 2% of mitoses, XY in 3% of mitoses)	Non smoker	33	Normal (at 27 years old)	9.8 (at time of DNA sampling)	2.5 (at time of DNA sampling)	None	Factor V Leiden, Disc disease (Scheuermann's disease)
2	30.33	47,XXY (XXY in 20% of mitoses, XX in 80% of mitoses)	Non smoker	31	NA	3.7 (at time of DNA sampling)	1.1 (at time of DNA sampling)	Antioxidant	Splenectomy at 6 years old
3	15.31	47,XXY (100% mitoses)	Non smoker	18	Osteopenia (at 20 years old)	6.5 (at 16 years old)	1.3 (at 16 years old)	Levetiracetam + sodium valproate	Epilepsy, varicocele
4	30.44	47,XXY (100% mitoses)	Ex smoker	23.5	NA	11.0 (at time of DNA sampling)	1.9 (at time of DNA sampling)	Testosterone gel since several years	Operated cryptorchidism, testosterone enanthate between 13 and 16 years old, testosterone undecanoate at 26 years old then testosterone gel
5	14.94	47,XXY (100% mitoses)	Smoker	27	Normal (at 16 years old), osteopenia at 20 years old	7.8 (at time of DNA sampling)	0.7 (at time of DNA sampling)	Testosterone enanthate since 6 months	None
6	15.40	47,XXY (100% mitoses)	Non smoker	19 (at 19 years old)	Osteopenia (at 18 years old)	8.3 (at time of DNA sampling)	1.3 (at time of DNA sampling)	None	None
7	15.97	47,XXY (100% mitoses)	Non smoker	19	NA	9.9 (at time of DNA sampling)	1.6 (at time of DNA sampling)	None	Autistic disorder, treated varicocele
8	30.38	47,XXY (100% mitoses)	Non smoker	21	NA	1.4 (at time of DNA sampling)	0.3 (at time of DNA sampling)	Testosterone undecanoate	Testosterone enanthate between 23 and 29 years old, then testosterone undecanoate
9	18.10	47,XXY (XXY in > 97% of mitoses, XY in < 3% of mitoses)	Smoker	19	NA	11.0 (at time of DNA sampling)	1.80 (at time of DNA sampling)	Abatacept + Rituximab	Juvenile rheumatoid arthritis treated by methotrexate and etanercept then tocilizumab until 14 years old, by abatacept and rituximab since 17 years old. Hodgkin lymphoma since 14 years old treated by chemotherapy and radiotherapy

Abbreviation: NA, not available.

These findings help explain why the GrimAge clock yields lower values in 47,XXY individuals compared to 46,XY individuals. One key factor is DNAmLeptin, which enters the model with a negative coefficient: higher DNAmLeptin in 47,XXY individuals reduces GrimAge relative to 46,XY men. Similarly, DNAmPACKYRS has a positive coefficient, so its lower values in 47,XXY individuals also contribute to reduced GrimAge. By contrast, DNAmADM, which also has a positive coefficient, does not explain the lower GrimAge in 47,XXY individuals, since its values are elevated rather than reduced. Taken together, DNAmLeptin and DNAmPACKYRS are the most likely drivers of the reduced age acceleration observed in 47,XXY individuals.

Finally, we note that DNAmTIMP1 was increased in 47,XYY compared to 46,XY males (*p* < 0.01, Figure 3C). TIMP1 is a regulator of extracellular matrix turnover, and elevated TIMP1 levels are often linked to fibrotic conditions (Egea et al. 2012; Hemmann et al. 2007; Todd et al. 2020).

### 2.4 DunedinPACE Corroborates the GrimAge Results

**FIGURE 4:**
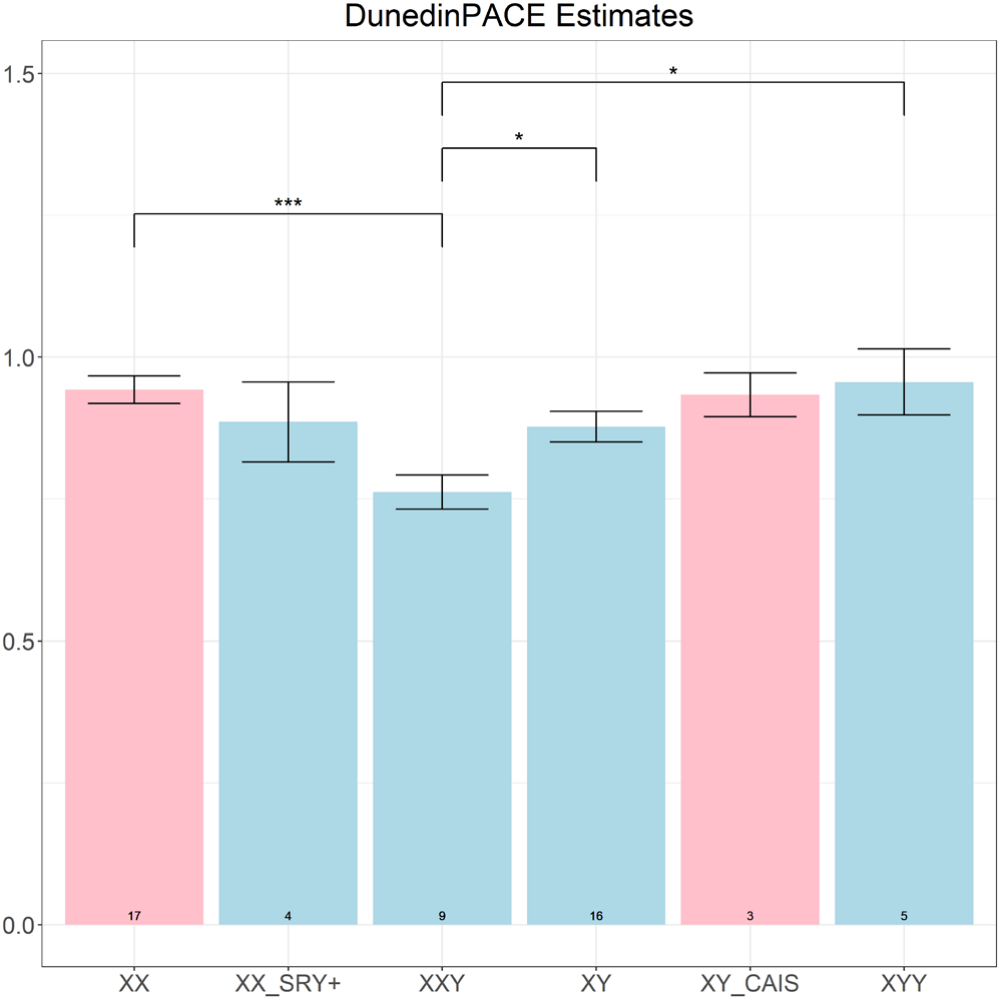
DunedinPACE estimates. The *y*‐axis signifies the estimated DunedinPACE. The groupings/bars correspond to XX females, XX SRY+ phenotypic males, XXY males, XY males, XY CAIS phenotypic females, and XYY males. The pink bars represent phenotypic females and the light blue bars represent phenotypic males. The significance brackets are displayed for select biologically relevant comparisons (see Section 5). The asterisks above the significance brackets correspond to significance levels from a Wilcoxon rank‐sum test of *p < 0.05, **p < 0.01, and ***p < 0.001. Each bar plot depicts the mean values (*y*‐axis) along with one standard error. Sample sizes (counts) are reported at the bottom of each bar.

The DunedinPACE clock indicates that 47,XXY males have a slower pace of aging than both 46,XY males (*p* < 0.05) and 46,XX females (*p* < 0.001). This pattern mirrors the GrimAge results, which show lower age acceleration in 47,XXY males relative to both comparison groups (Figure 4). Additionally, 47,XXY males have a slower pace of aging than 47,XYY males (*p* < 0.05).

### 2.5 Blood Cell Counts Associated With T Cell Senescence

**FIGURE 5:**
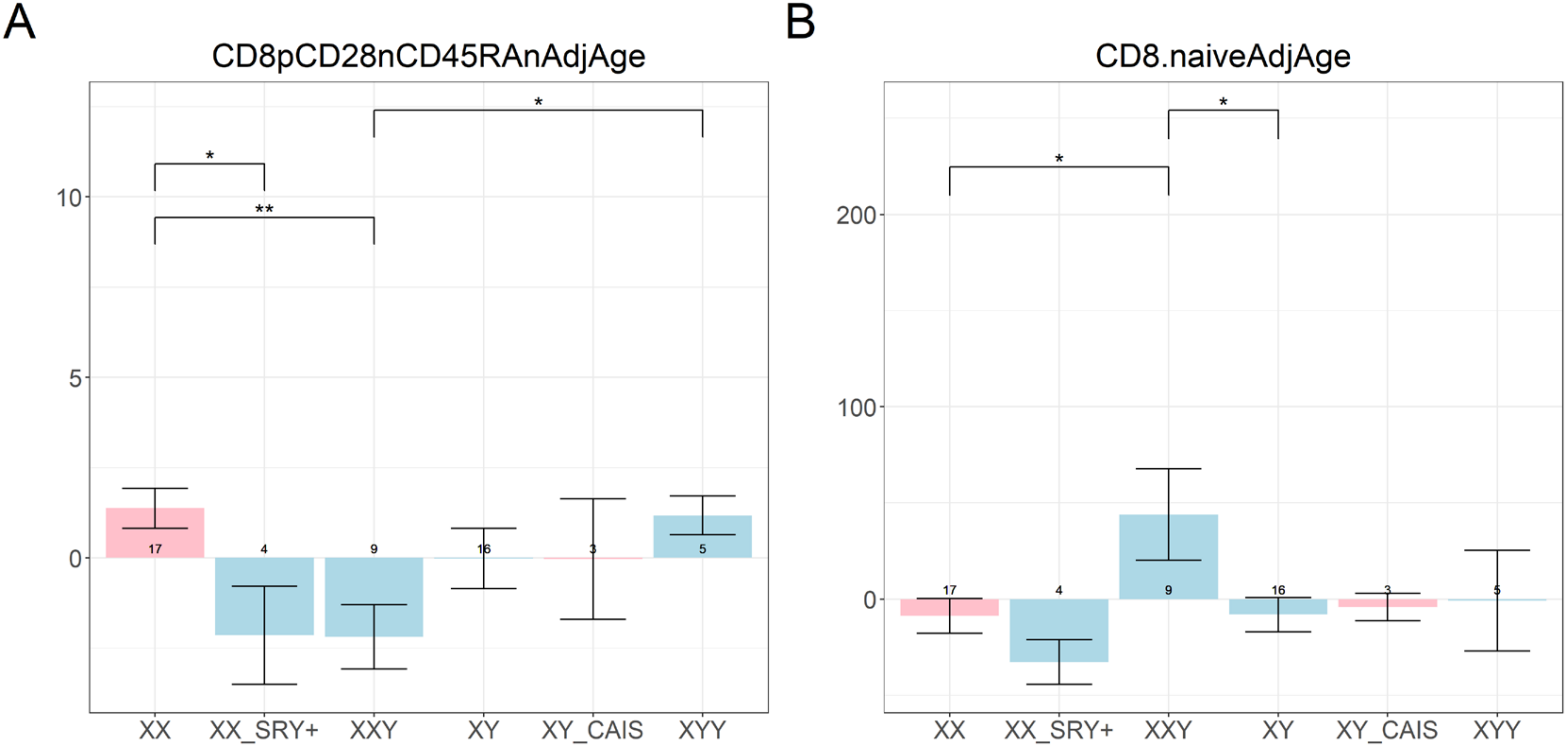
Estimated abundance measures of blood cells. The *y*‐axis signifies the estimated abundance of (A) exhausted CD8^+^ T cells and (B) naïve CD8^+^ T cells. The groupings/bars correspond to XX females, XX SRY+ phenotypic males, XXY males, XY males, XY CAIS phenotypic females, and XYY males. The pink bars represent phenotypic females and the light blue bars represent phenotypic males. The significance brackets are displayed for select biologically relevant comparisons (see Section 5). The asterisks above the significance brackets correspond to significance levels from a Wilcoxon rank‐sum test of *p < 0.05, **p < 0.01, and ***p < 0.001. Each bar plot depicts the mean values (*y*‐axis) along with one standard error. Sample sizes (counts) are reported in each bar. These DNAm‐derived measures reflect ordinal cell abundance and should not be interpreted as absolute cell counts or percentages.

We estimated two cytotoxic T cell measures from DNAm: exhausted CD8^+^ (CD8^+^CD28^−^CD45RA^−^AdjAge) and naïve CD8^+^ T cells (Figure 5) based on Horvath et al. (2016). After adjusting for chronological age, estimated exhausted CD8^+^ T cell abundance did not differ between 47,XXY and 46,XY, whereas estimated naïve CD8^+^ T cells were higher in 47,XXY (*p* < 0.05), consistent with a younger cytotoxic T cell compartment (Figure 5). Overall, this suggests that the cytotoxic CD8^+^ T cells of 47,XXY individuals are biologically younger than those of 46,XY individuals.

### 2.6 EWAS of Karyotype

Many CpGs on the X chromosome were hypermethylated in individuals with more than one X chromosome, reflecting the effects of X inactivation (Figure S1). To avoid this confounding effect, we focused our analyses on autosomal CpGs. Using linear regression models, we performed epigenome‐wide association studies (EWAS) of autosomal CpG methylation while adjusting for age. Three comparisons were examined: (i) 47,XXY versus 46,XY (Figures S2 and S3), (ii) 47,XXY versus 46,XX (Figures S4 and S5), and (iii) 47,XYY versus 46,XY (Figures S6 and S7). For functional enrichment analyses, we used the GREAT tool. In our experience, it is valuable to distinguish between hypermethylation and hypomethylation. Accordingly, we selected the 4000 most significant CpGs from each EWAS and divided them into the 2000 most strongly hypermethylated sites (positive associations) and the 2000 most strongly hypomethylated sites (negative associations) for input into GREAT.

GREAT analysis of CpGs differentially methylated in 47,XXY compared to 46,XY revealed enrichment of immune‐related pathways. Hypomethylated CpGs were associated with genes involved in lymphocyte activation and T cell differentiation, as well as autoimmune disease signatures such as systemic lupus erythematosus (FDR = 8.4 × 10^−9^). Additional enrichments included cadherin signaling and developmental abnormalities such as Arnold–Chiari malformation. By contrast, hypermethylated CpGs were enriched for genes linked to neurological and hematological conditions (e.g., restless legs syndrome, hemihypertrophy), cell cycle regulation (aurora B signaling, stathmin family), and cancer‐related pathways (acute promyelocytic leukemia, Huntington disease). These results suggest that 47,XXY is characterized by methylation changes in immune regulatory genes as well as loci relevant to cell cycle control and neurological function.

When comparing 47,XXY to 46,XX individuals, hypomethylated CpGs again showed strong enrichment for immune activation pathways, including T cell activation, chemokine receptor signaling, and abnormal T cell numbers (FDR = 6.2 × 10^−19^). The most striking enrichments were autoimmune‐related signatures (e.g., systemic lupus erythematosus CD4 T cells vs. B cells; FDR = 1.4 × 10^−68^), underscoring a consistent association between 47,XXY and altered immune epigenetic profiles. Hypermethylated CpGs, in contrast, were enriched for metabolic and cancer‐related processes, including type II diabetes, adenocarcinoma, protein metabolism, and mRNA splicing. Taken together, these findings indicate that 47,XXY is associated with a dual pattern of altered methylation, involving both upregulation of immune signaling pathways and shifts in metabolic and oncogenic pathways.

When comparing 47,XYY to 46,XY individuals, hypomethylated CpGs were enriched for genes involved in amino acid transport and metabolism, as well as immune responses such as LPS‐stimulated monocyte activation and interferon‐related pathways. Hypermethylated CpGs, on the other hand, highlighted processes related to renal dysfunction (abnormal renal morphology, chronic transplant rejection), immune dysregulation (HIV infection pathways, B cell subsets), and FGF signaling. Thus, while 47,XYY shares with 47,XXY a pattern of immune‐related epigenetic changes, it also displays distinct enrichments in renal and metabolic pathways.

Overall, these GREAT analyses reveal that both 47,XXY and 47,XYY are marked by widespread immune‐related epigenetic alterations, particularly involving T cell function and autoimmunity. At the same time, the broader biological consequences appear to diverge: 47,XXY shows stronger connections to metabolic and cancer‐associated pathways, whereas 47,XYY is more strongly linked to renal biology and amino acid metabolism. These pathway enrichments align with known clinical differences between sex chromosome aneuploidies: 47,XXY is associated with increased autoimmune, metabolic, thrombotic, and specific cancer risks, whereas 47,XYY shows heightened respiratory and neurodevelopmental morbidity with broadly elevated disease burden (Davis et al. 2020; Scofield et al. 2008). Accordingly, the distinct enrichment profiles observed here may help explain differences in health risks across karyotypes; renal pathway signals in 47,XYY should only be considered hypothesis‐generating pending stronger epidemiological confirmation.

## 3 Discussion

Using next‐generation DNA methylation (DNAm) clocks, we observed that 47,XXY individuals exhibit slower epigenetic aging than both 46,XY and 46,XX: the GrimAge clock showed lower age acceleration, and DunedinPACE indicated a slower pace of aging in 47,XXY. By contrast, the first‐generation Skin & Blood clock indicated higher age acceleration in 47,XXY and 47,XYY relative to 46,XY. These divergent patterns reflect that different clocks capture distinct domains of epigenetic aging.

GrimAge is linked to immunosenescence (Lu, Quach, et al. 2019). Consistent with slower immune aging, 47,XXY individuals showed higher estimated naïve CD8^+^ T cell abundance, a subset that declines with age (Fagnoni et al. 2000; Lazuardi et al. 2005) and contributes to immune aging (Goronzy et al. 2015). Prior work also reported higher CD8^+^ T cell counts in 47,XXY versus 46,XY (Koçar et al. 2001). Multi‐omic profiling demonstrated broad differences between 47,XXY and 46,XY, including X‐linked hypermethylation and immune‐gene dysregulation (Skakkebæk et al. 2018). Overexpression of X‐linked *TLR7* and *CD40L* in healthy females and 47,XXY patients supports an X‐dose effect on innate and adaptive responses (Sarmiento et al. 2019). The more robust immune tone in females comes with greater autoimmunity; a similar tendency in 47,XXY may help explain their predisposition to autoimmune disease (Sarmiento et al. 2019). Moreover, 47,XXY patients show lower cytokine inflammatory responses in whole blood, with a female‐like production pattern that persists after adjusting for sex steroids—implicating sex chromosomes more than hormones in cytokine profiles (Lefèvre et al. 2019). PAI‐1 is linked to pro‐inflammatory states (Morrow et al. 2021), and its DNAm surrogate associates with inflammation (Lu, Quach, et al. 2019); we found suggestive evidence for lower DNAmPAI1 in 47,XXY versus 46,XY (*p* = 0.09). Clinical records indicated no osteoporosis in most patients, compatible with preserved immune–bone crosstalk (Madel et al. 2019; Terashima and Takayanagi 2019), though this requires confirmation.

Telomere findings are mixed across studies. Young 47,XXY patients (18–24 years) can have longer telomeres, a difference that may diminish with age (Pohl et al. 2020); telomere attrition has been implicated in B cell acute lymphoblastic leukemia (Kjeldsen 2022). In our 14.9–30.4‐year‐old cohort, DNAm‐based telomere length was longer in 47,XXY than 46,XY, an effect that may reflect age range. In general, DNAmTL differs from actual telomere length (based on PCR measurements or Southern blotting). DNAmTL correlates only moderately with actual leukocyte telomere length (*r* ≈0.4) (Lu, Seeboth, et al. 2019).

Slightly reduced lifespan (~2 years) and broad comorbidity burden can be observed in 47,XXY men including infectious and autoimmune disease risk, breast and mediastinal tumors, endothelial dysfunction, cardiac abnormalities including short QTc, low bone density/osteoporosis, tic disorders, dyslipidemia and obesity, diabetes, cognitive and neuroanatomical differences, hypogonadism/azoospermia, reduced muscle strength, and delayed motor development (Bojesen et al. 2004, 2006; Bojesen and Gravholt 2011; Belling et al. 2017; Kanakis and Nieschlag 2018; Davis et al. 2024). Unexpectedly, our epigenetic results indicate slower epigenetic aging in 47,XXY men compared to 46,XY men. A recent study reported a lower estimated 10‐year survival (Charlson Comorbidity Index) in 47,XXY compared with matched 46,XY controls but no difference in age at death (Davis et al. 2024). This apparent paradox may arise if mortality is driven by factors outside what GrimAge/DunedinPACE capture, for example, socioeconomic determinants with > 10‐year life‐expectancy gaps in the United States (Singh and Lee 2021). Additional biological hypotheses include mitigation of the “unguarded X” effect by an extra X (Xirocostas et al. 2020; Connallon et al. 2022); resource‐allocation/reproductive‐cost differences given sterility (Jasienska 2020; Fuchs et al. 2021) and parallels with reduced androgen exposure (Gems 2014), longer lifespan after sterilization in dogs (Hoffman et al. 2013), and slower epigenetic aging after castration in sheep (Sugrue et al. 2021); and viability selection favoring a lower deleterious‐variant burden among surviving 47,XXY (Popadin et al. 2018). These findings remain hypothesis‐generating.

We also note that 47,XYY showed the highest acceleration on the Skin & Blood clock, underscoring that first‐generation clocks estimating chronological age and next‐generation clocks linked to morbidity/mortality may diverge and should be interpreted in light of their distinct definitions.

### 3.1 Limitations

Some biomarkers evaluated here such as GrimAge and DNAmLeptin include X chromosome CpGs (Table S1). Because many X‐linked CpGs are hypermethylated when the karyotype includes > 1 X chromosome due to X inactivation (Figure S1), such models could, in principle, introduce artefactual associations in 47,XXY. DunedinPACE, which excludes X‐linked CpGs (Table S1), nevertheless showed the same direction of effect (47,XXY < 46,XY), partially mitigating this concern. More broadly, these clocks were trained largely in euploid populations, so calibration and absolute estimates may differ in sex chromosome aneuploidies.

The study population was predominantly young and cross‐sectional; however, prior work on young adults was able to highlight EAA (Foster et al. 2023). Nevertheless, we cannot determine whether GrimAge/DunedinPACE differences in 47,XXY versus 46,XY persist, attenuate, or reverse at older ages. Divergent signals across clocks in our dataset (lower GrimAge and DunedinPACE vs. higher Skin & Blood in 47,XXY) underscore the need for longitudinal, age‐spanning studies.

Sample size limited statistical power and covariate control. Larger cohorts should more fully model BMI (Foster et al. 2023), social determinants (Aiello et al. 2024), testosterone and other sex steroids (Lefèvre et al. 2019), medical history, smoking (status, intensity, duration), and medication/treatment exposures (including testosterone dose and duration). Differences in smoking prevalence may leave residual confounding even when using DNAmPACKYRS.

Ascertainment and survivorship bias are possible: the 47,XXY group comprises clinically diagnosed individuals seeking fertility preservation and may not represent undiagnosed 47,XXY. Clinical ascertainment correlates with outcomes (diagnosed 47,XXY show higher estimated 10‐year survival than undiagnosed 47,XXY; Davis et al. 2024), potentially biasing effect estimates.

Our analyses used blood, and DNAm is tissue specific. Immune‐pathway enrichments may reflect regulatory changes and cell‐composition differences; we relied on DNAm‐based cell estimates rather than direct cytometry. Likewise, DNAm surrogates for proteins (e.g., leptin, PAI‐1) were not validated against plasma measurements in this cohort, which should be done in future studies. Technical/analytical factors (e.g., platform and normalization choices) and potential batch effects may also add noise. Gene‐set enrichment (e.g., GREAT) depends on the background, ontology curation, and our top‐CpG thresholding (2000 hyper/2000 hypo), so pathway results should be viewed as hypothesis‐generating.

We did not survey additional epigenetic clocks due to space constraints. Our 46,XY versus 47,XXY findings differ from a larger study using a three‐CpG clock that reported no acceleration (Pohl et al. 2020).

GrimAge is not equivalent to mortality risk. While the two are significantly associated, they represent distinct measures of organismal aging. Epigenetic biomarkers such as GrimAge capture only one dimension among the many biological and clinical indicators that contribute to mortality risk. Overall, our molecular profiling focused on DNAm clocks and DNAm‐based cell estimates; we did not evaluate alternative aging biomarkers (e.g., proteomic, mitochondrial, metabolomic, glycomic, or immune functional measures), which will be important for integrative, mechanistic inference.

## 4 Conclusion

In summary, 47,XXY showed lower GrimAge acceleration and slower DunedinPACE, as well as longer DNAm‐based telomere estimates, relative to 46,XY. Higher naïve CD8^+^ T cell estimates support reduced immunosenescence in 47,XXY. We hypothesize that X chromosome dosage and downstream immune and hormonal effects contribute to these patterns; nevertheless, larger, mechanistic, and longitudinal studies are needed to reconcile clock divergence with clinical outcomes and to define how sex chromosomes shape human aging.

## 5 Methods

### 5.1 Biological Material

The study was approved by the ethical committee of Lyon University Hospital (number of the ethical committee: 22_385, number in the register of the Commission nationale de l'informatique et des libertés: 22_5385).

Fifty‐four individuals were included (Table 1). Thirty‐three had a normal chromosomal formula (17 women with a 46,XX karyotype and 16 men with a 46,XY karyotype); they were asymptomatic partners of an individual affected by a relatively frequent autosomal recessive disease (cystic fibrosis or congenital adrenal hyperplasia). Fourteen individuals had sex chromosome aneuploidy (nine men with a 47,XXY karyotype, five men with a 47,XYY karyotype), four individuals had a 46,XX SRY positive karyotype leading to a male phenotype, and three were 46,XY with a mutated androgen receptor leading to a complete androgen insensitivity syndrome characterized by a female phenotype (46,XY CAIS). The 47,XXY, 47,XYY, 46,XX SRY positive, and 46,XY CAIS individuals were recruited in the fertility preservation or genetic unit of the Lyon University Hospital. The age of the individuals ranged from 14 to 39, and 14 out of 54 were current smokers.

EDTA blood samples were collected and DNA was extracted using the Nucleospin Blood L Vacuum kit from Macherey‐Nagel (Hoerdt, France) in a microlab STARlet extractor (Hamilton, Nevada, USA). DNA extracts were kept at 4°C until analysis. The concentration of DNA was 259 ng/μL in mean (range: 102–518) using a fluorometric measurement with Quant‐iT 1X ds DNA HS Assay kit (ThermoFisher Scientific, Massachusetts, USA) and EnSpire Plate Reader (PerkinElmer, Massachusetts, USA). Some extracts were used several times (replicates) (Table 1) to prepare a 96‐well plate of 25 μL of 70 ng/μL DNA. Replicates were averaged in subsequent downstream analyses.

### 5.2 Methylation Profiling

The DNA methylation data were generated using the Illumina MethylationEPIC Beadchip (Illumina Inc., California, USA). The methylation data were normalized using the “noob” normalization method implemented in the minfi R package (Aryee et al. 2014). The beta values for the technical replicates were averaged prior to further analyses to create one methylation profile for each individual. The CpGs were separated into CpGs located on autosomes (*n* = 846,232), CpGs located on the X chromosome (*n* = 19,090), and CpGs located on the Y chromosome (*n* = 537).

### 5.3 Epigenetic Clocks

We utilized the online epigenetic clock software to compute all DNAm‐based biomarkers aside from DunedinPACE (available at https://dnamage.clockfoundation.org/calculator). For the GrimAge clock, which is a composite biomarker that includes sex, we set the sex of the samples as female to ensure consistent results in consideration of the multiple types of aneuploidies. We focused on nine biologically relevant comparisons of karyotypes, comprising of the following: 46,XX versus 46,XY, 46,XY CAIS versus 46,XY, 46,XY versus 47,XYY, 46,XX versus 47,XXY, 46,XY versus 47,XXY, 46,XX versus 46,XX SRY+, 46,XX versus 47,XYY, 46,XX versus 46,XY CAIS, and 47,XYY versus 47,XXY.

First‐generation clocks, like the pan‐tissue clock by Horvath (2013), the skin and blood clock by Horvath et al. (2018), and the blood‐based clock by Hannum et al. (2013), primarily gauge chronological age. In contrast, next‐generation clocks focus on assessing mortality risk. Examples include DNAm PhenoAge by Levine et al. (2018) and DNAm GrimAge (Lu, Quach, et al. 2019). The DNAm GrimAge is a prominent estimator of human mortality risk based on DNA methylation (DNAm). It stands as one of the most recognized DNAm aging biomarkers (Lu, Quach, et al. 2019). There are also clocks estimating telomere length (Lu, Seeboth, et al. 2019). The “age acceleration” was calculated by taking the raw residual of the epigenetic aging biomarker, or the predicted age, and regressing it upon chronological age.

DunedinPACE is a next‐generation epigenetic clock that seeks to measure the pace of aging (Belsky et al. 2022). Rather than estimating an age, DunedinPACE estimates a value that represents the number of years of biological aging for each year of chronological age, such that a value of approximately 1 is expected for midlife adults.

### 5.4 Epigenome‐Wide Association Study (EWAS)

EWAS was conducted by performing a linear regression where the CpG beta values were regressed upon the karyotype and age of the samples. We performed comparisons of karyotypes where each karyotype was coded as either 0 or 1: 46,XX (0) versus 47,XXY (1), 46,XY (0) versus 47,XXY (1), and 46,XY (0) versus 47,XYY (1).

### 5.5 Genomic Regions Enrichment of Annotations Tool (GREAT)

GREAT analysis was conducted using the rGREAT R package (Gu and Hübschmann 2023). GREAT performs hypergeometric tests using a foreground and background set and returns annotations of gene sets near the foreground CpGs. The foreground set consisted of 4000 CpGs in total, taking the top 2000 most significantly positively and negatively associated autosomal CpGs from the EWAS comparisons of karyotype. The background set consisted of all the autosomal CpGs on the EPIC array. In total, 18 sets of GREAT analyses were performed between nine different comparisons and two sets of positively and negatively associated CpGs for each comparison. All reported ontologies have an FDR‐adjusted hypergeometric *p* value < 0.05 and an unadjusted hypergeometric *p* value < 0.001. The GREAT settings used were hg19 for the species assembly, proximal: 5.0 kb upstream and 1.0 kb downstream, distal: 50 kb.

## Author Contributions

Conceptualization: G.A.B.M., I.P., C.V., J.‐F.L.; Methodology: S.H., G.A.B.M., B.R., I.P., C.V., J.‐F.L.; Software: S.H., J.Z.; Validation: J.Z., S.H.; Formal analysis: J.Z., J.T.; Investigation: J.Z., J.T., J.‐F.L., C.V., I.P., G.A.B.M., S.H.; Resources: J.T., I.P., S.H., B.R.; Data curation: J.Z.; Writing – original draft: G.A.B.M., S.H., J.Z., J.T.; Writing – review and editing: all authors; Visualization: J.Z., J.T.; Supervision: S.H., I.P., G.A.B.M.; Project administration: G.A.B.M., I.P., S.H.; Funding acquisition: G.A.B.M., I.P., C.V., J.‐F.L., S.H.

## Conflicts of Interest

The Regents of the University of California are the sole owner of patents and patent applications directed at epigenetic biomarkers (including GrimAge, PhenoAge, Skin & Blood clock, telomere length) for which Steve Horvath is a named inventor; S.H. is a founder and paid consultant of the nonprofit Epigenetic Clock Development Foundation that licenses these patents. S.H. is a Principal Investigator at the Altos Labs, Cambridge Institute of Science, a biomedical company that works on rejuvenation. The other authors declare no competing interests.

## Supporting information


**Appendix S1:** acel70243‐sup‐0001‐AppendixS1.docx.

## Data Availability

The data that support the findings of this study are available on request from the corresponding author. The data are not publicly available due to privacy or ethical restrictions.
