# Supplementary material for: Epigenetic Clock Analysis of Sex Chromosome Aneuploidies
Source: Aging Cell. 2025 Sep 30;24(11):e70243. doi: 10.1111/acel.70243 (PMC12610412; doi:10.1111/acel.70243)
Supplement: Supplementary file 1 — Appendix S1: acel70243‐sup‐0001‐AppendixS1.docx. [file ACEL-24-e70243-s001.docx]

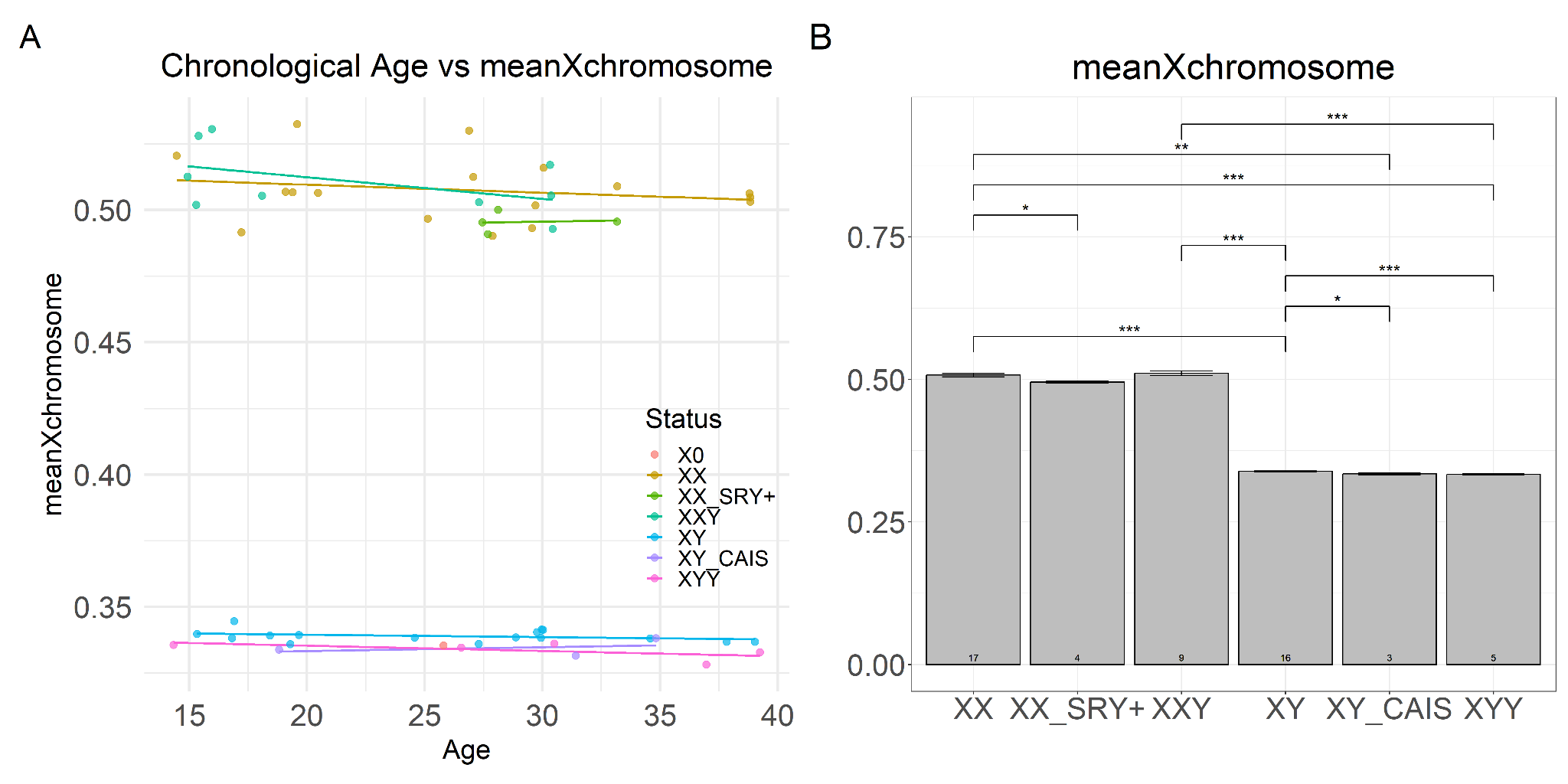


**Supplementary** **Figure S1**: **Mean methylation on X chromosome.** **(A)** Chronological age versus mean methylation on the X chromosome, where age is measured in calendar years. **(B)** Mean methylation on the X chromosome stratified by karyotype. The groupings/bars correspond to XX females, XX SRY+ phenotypic males, XXY males, XY males, XY CAIS phenotypic females, and XYY males. The significance brackets are displayed for select biologically relevant comparisons (see Materials and methods section). The asterisks above the significance brackets correspond to significance levels from a Wilcoxon rank-sum test of p < 0.05 (*), p < 0.01 (**), and p < 0.001 (***). Each bar plot depicts the mean values (y-axis) along with one standard error. Sample sizes (counts) are reported in each bar.


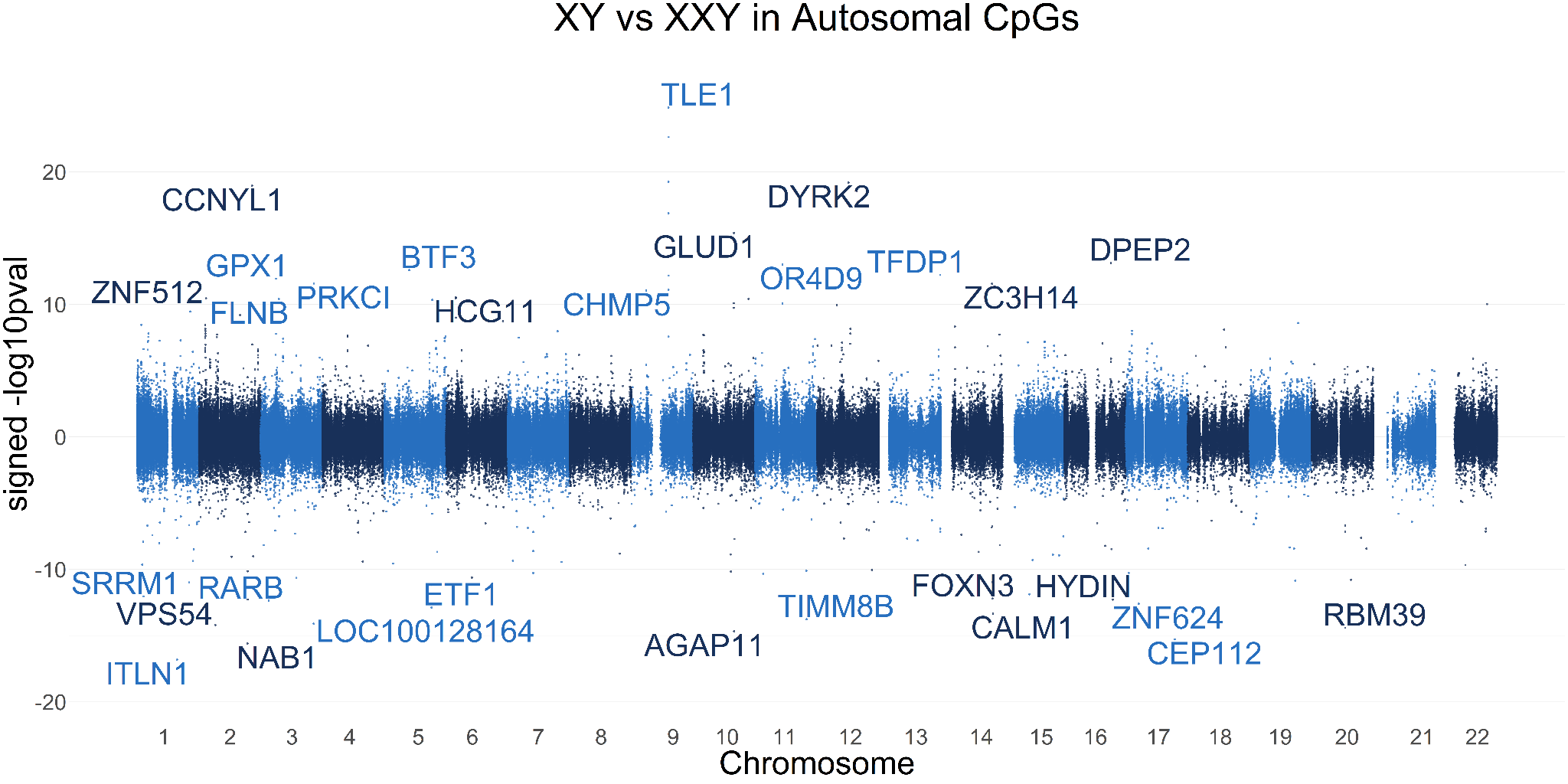


**Supplementary Figure S2: Manhattan Plot of EWAS of differentially methylated autosomal CpGs in 46,XY compared to 47,XXY.** Top CpGs are labeled with the names of proximal genes. The x-axis represents the location of the CpG on the chromosome and the y-axis represents the signed -log10 p-value. The positive y-values are genes associated with CpGs that have greater methylation in 47,XXY compared to 46,XY, while the negative y-values are genes associated with CpGs that have lower methylation in 47,XXY compared to 46,XY.


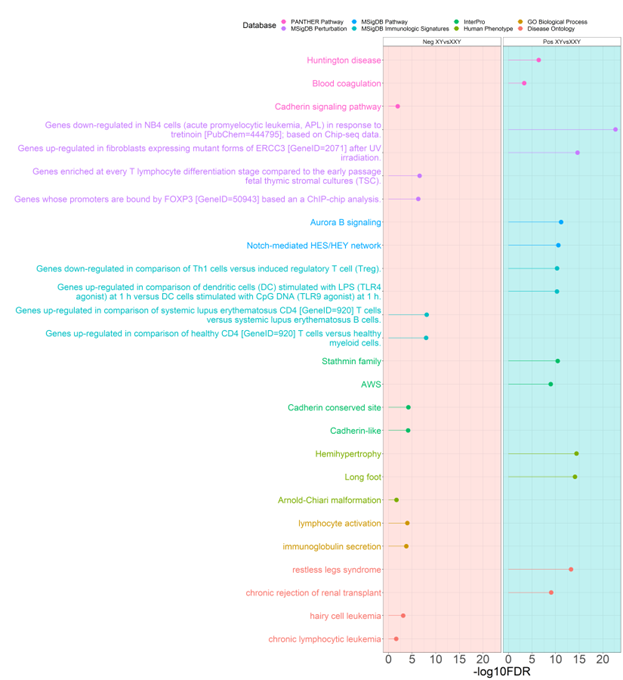


**Supplementary Figure S3: Enrichment analysis of EWAS comparing 46,XY males to 47,XXY males.** Analysis focused on the top 2,000 CpGs with increased methylation in 47,XXY males relative to 46,XY males (pos) and the top 2,000 CpGs with decreased methylation in 47,XXY males relative to 46,XY males (neg)**.** Represented here are up to two of the most significant ontologies from each ontology database for each positive and negative set of autosomal CpGs. The background shaded in red indicates the negative set, while the background shaded in blue indicates the positive set. The x-axis represents the -log10 of FDR-adjusted hypergeometric p-values. All results fall below an FDR-adjusted p-value threshold of <0.05.

**
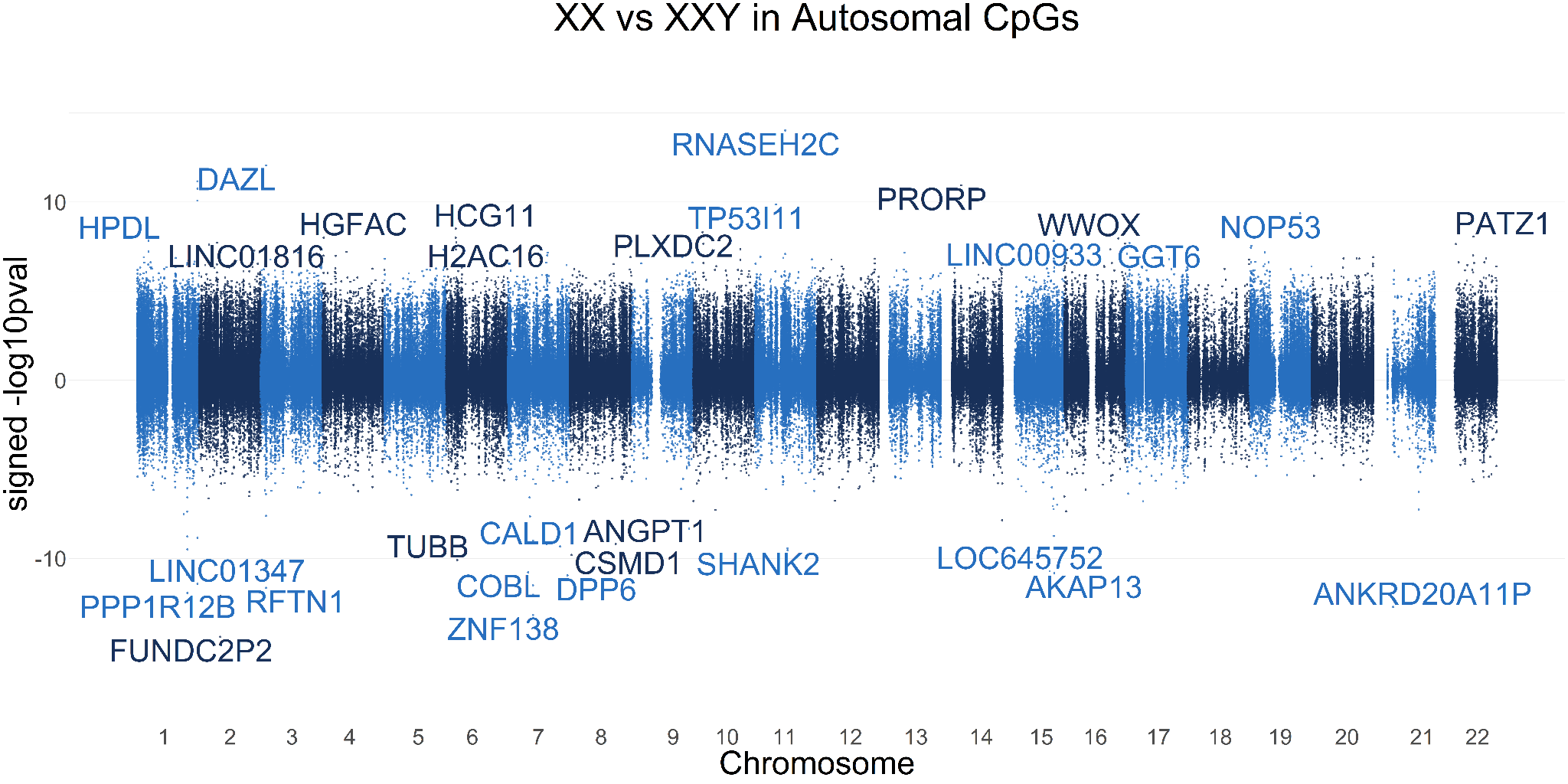
Supplementary Figure S4: Manhattan Plot of EWAS of differentially methylated autosomal CpGs in 46,XX compared to 47,XXY.** Top CpGs are labeled with the names of proximal genes. The x-axis represents the location of the CpG on the chromosome and the y-axis represents the signed -log10 p-value. The positive y-values are genes associated with CpGs that have greater methylation in 47,XXY compared to 46,XX, while the negative y-values are genes associated with CpGs that have lower methylation in 47,XXY compared to 46,XX.


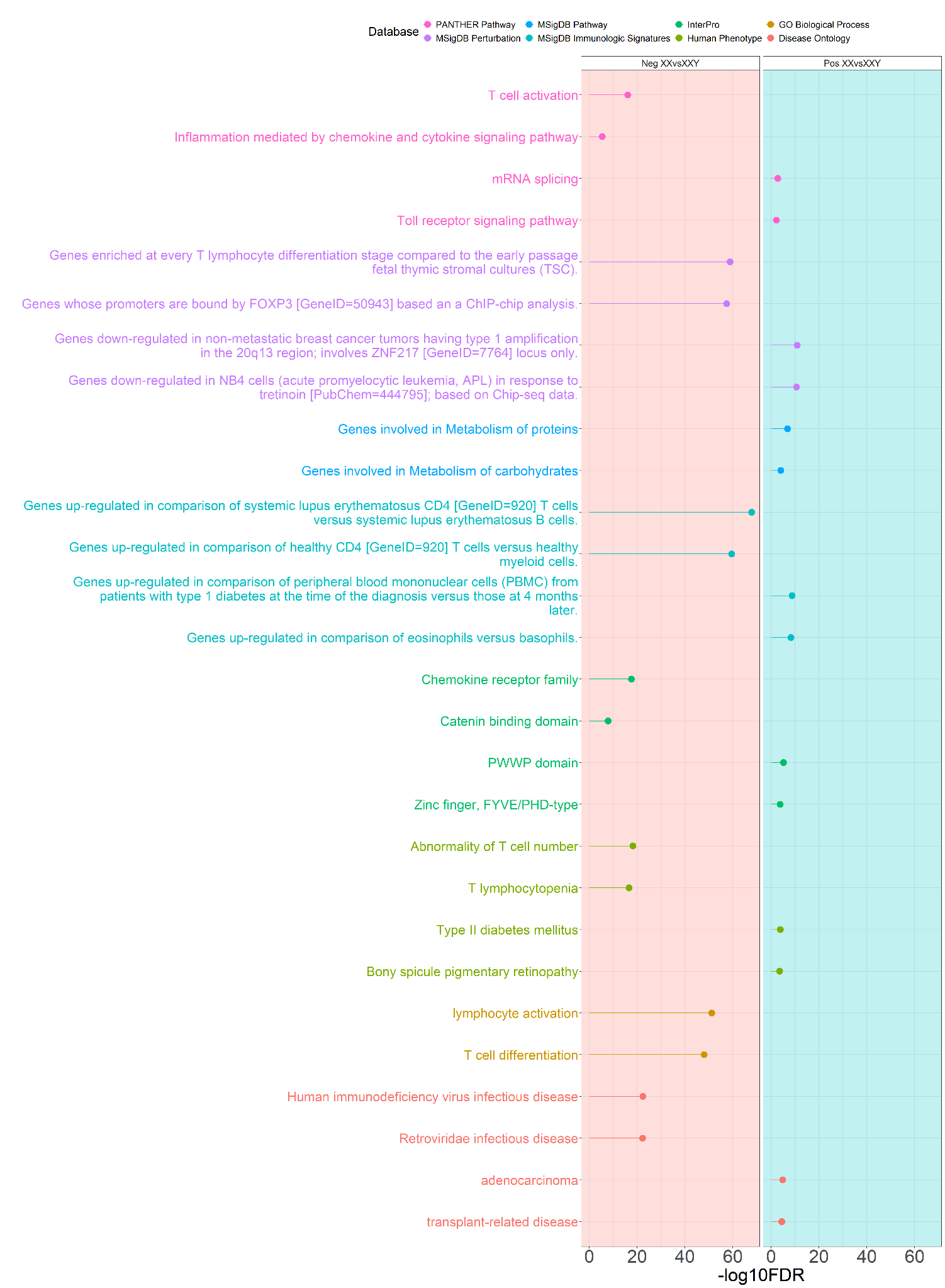


**Supplementary Figure S5: Enrichment analysis of EWAS comparing 46,XX females to 47,XXY males.** Analysis focused on the top 2,000 CpGs with increased methylation in 47,XXY males relative to 46,XX females (pos) and the top 2,000 CpGs with decreased methylation in 47,XXY males relative to 46,XX females (neg)**.** Represented here are up to two of the most significant ontologies from each ontology database for each positive and negative set of autosomal CpGs. The background shaded in red indicates the negative set, while the background shaded in blue indicates the positive set. The x-axis represents the -log10 of FDR-adjusted hypergeometric p-values. All results fall below an FDR-adjusted p-value threshold of <0.05.


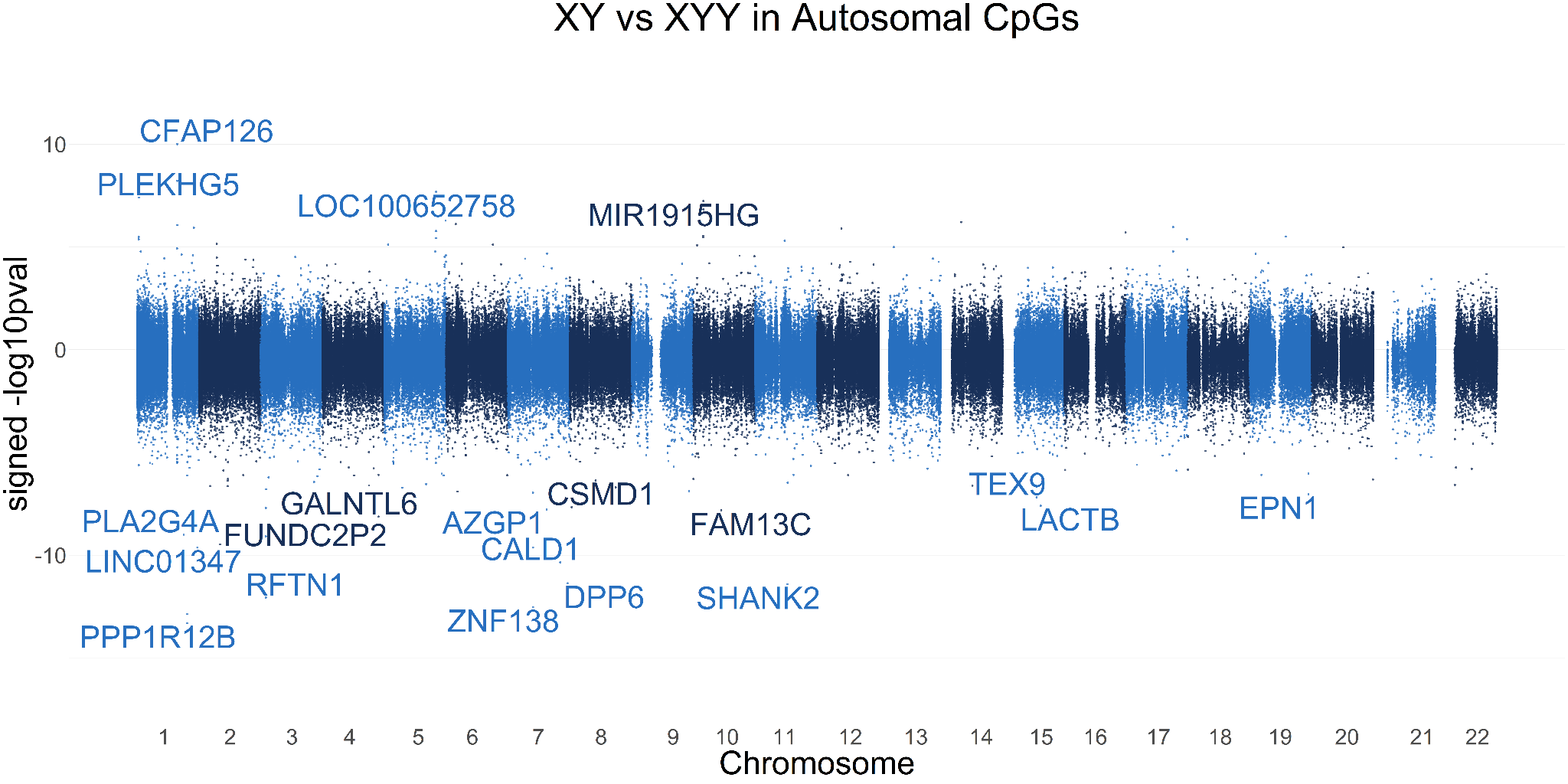


**Supplementary Figure S6: Manhattan Plot of EWAS of differentially methylated autosomal CpGs in 46,XY compared to 47,XYY.** Top CpGs are labeled with the names of proximal genes. The x-axis represents the location of the CpG on the chromosome and the y-axis represents the signed -log10 p-value. The positive y-values are genes associated with CpGs that have greater methylation in 47,XYY compared to 46,XY, while the negative y-values are genes associated with CpGs that have lower methylation in 47,XYY compared to 46,XY.


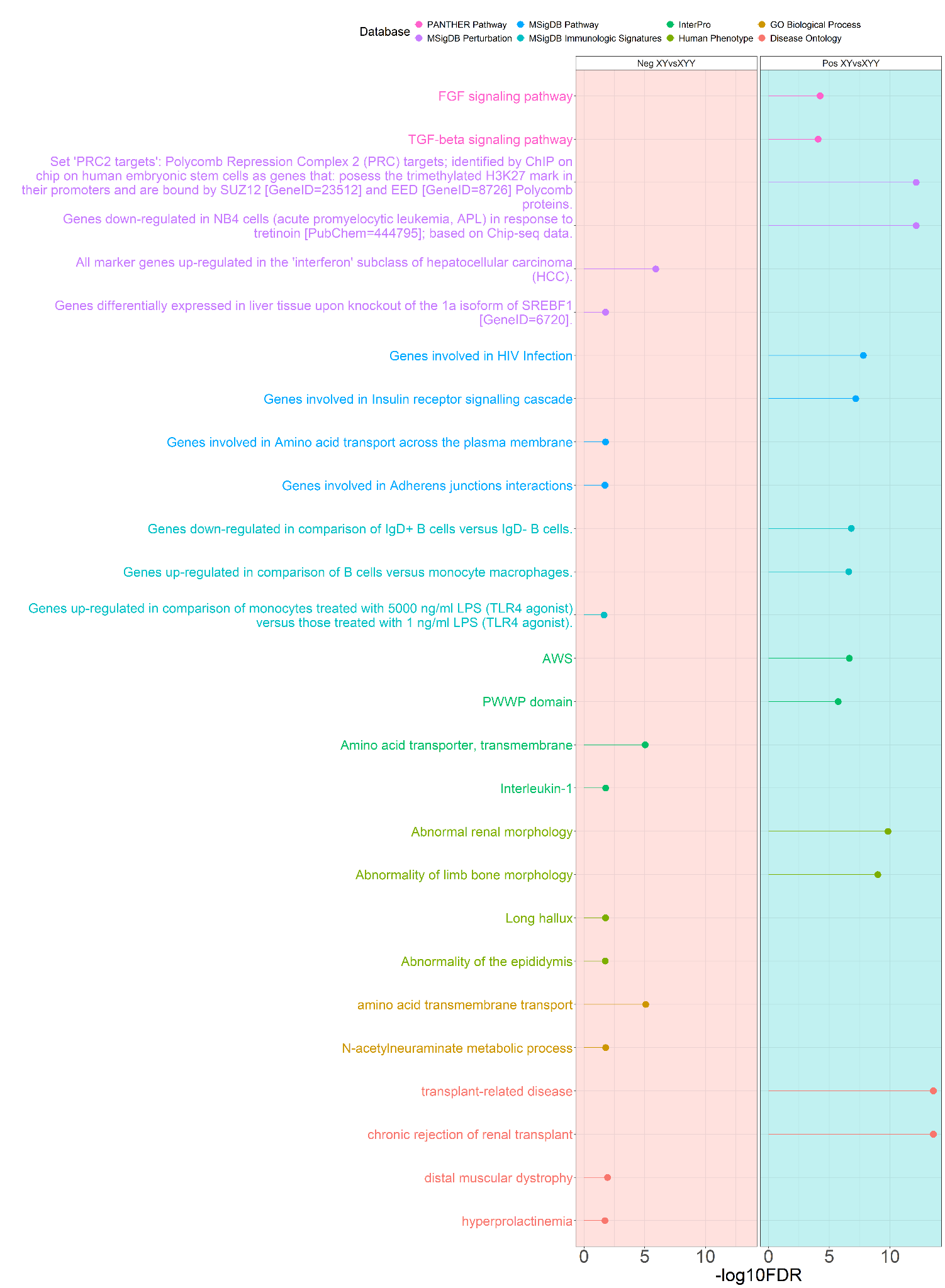


**Supplementary Figure S7: Enrichment analysis of EWAS comparing 46,XY males to 47,XYY males.** Analysis focused on the top 2,000 CpGs with increased methylation in 47,XYY males relative to 46,XY males (pos) and the top 2,000 CpGs with decreased methylation in 47,XYY males relative to 46,XY males (neg)**.** Represented here are up to two of the most significant ontologies from each ontology database for each positive and negative set of autosomal CpGs. The background shaded in red indicates the negative set, while the background shaded in blue indicates the positive set. The x-axis represents the -log10 of FDR-adjusted hypergeometric p-values. All results fall below an FDR-adjusted p-value threshold of <0.05.

| **Epigenetic Clock** | **CpGs on X Chromosome out of total number of CpGs in clock** |
| --- | --- |
| DNAmADM | 23/188 |
| DNAmB2M | 0/93 |
| DNAmCystatinC | 0/89 |
| DNAmGDF15 | 1/139 |
| DNAmLeptin | 32/188 |
| DNAmPACKYRS | 3/174 |
| DNAmPAI1 | 10/212 |
| DNAmTIMP1 | 2/44 |
| DunedinPACE | 0/173 |

**Supplementary Table S1: X-chromosome CpG content of DNAm biomarkers.** For each GrimAge component and DunedinPACE, the table reports the number of CpGs located on the X chromosome and the total number of CpGs used in the biomarker model (reported as X-chromosome CpGs / total CpGs).
